# Retrospective Analysis of Efficacy and Toxicity of Low-Dose Weekly Cisplatin Concurrently with Radiotherapy for p16-Positive Oropharyngeal Squamous Cell Carcinoma

**DOI:** 10.3390/cancers18203308

**Published:** 2026-10-14

**Authors:** Nitin Niranjan, Zulfiquer Otty, Abhishek Joshi, Marcus Hu, Oskar Edkins, Sarah Deacon, Courtney Fisher, Amy Brown, Corinne Ryan, Nathan Bain, Joanne Tan, Timothy Squire, Simon Liebenberg, Sabe Sabesan, Daniel Tao Xing, Shivanshan Pathmanathan

**Affiliations:** 1 Department of Radiation Oncology, Townsville University Hospital, Townsville, QLD 4814, Australia; nitin.niranjan@health.qld.gov.au (N.N.); timothy.squire@health.qld.gov.au (T.S.); 2 School of Medicine and Dentistry, Griffith University, Gold Coast, QLD 4222, Australia; 3 Department of Medical Oncology, Townsville University Hospital, Townsville, QLD 4814, Australia; 4 College of Medicine and Dentistry, James Cook University, Townsville, QLD 4814, Australia; 5 Department of Otolaryngology Head and Neck Surgery, Townsville University Hospital, Townsville, QLD 4814, Australia; 6 Department of Nutrition and Dietetics, Townsville University Hospital, Townsville, QLD 4814, Australia; 7 Department of Speech Pathology, Townsville University Hospital, Townsville, QLD 4814, Australia; 8 Australian Tropical Institute of Health and Medicine, James Cook University, Townsville, QLD 4814, Australia

**Keywords:** cisplatin, HPV, oropharyngeal squamous cell carcinoma, radiotherapy, survival

## Abstract

Background: The incidence of p16-positive oropharyngeal squamous cell carcinoma (OPSCC) is increasing, with improved prognosis compared to non-HPV-related cancers. Standard treatment of locally advanced OPSCC involves high-dose cisplatin (HDC) with radiation but is associated with significant toxicity. Low-dose cisplatin (LDC) administered weekly has shown comparable efficacy to HDC in prior studies. This study evaluates the real-world outcomes of LDC in p16-positive OPSCC in a regional centre. Material and methods: Patients treated with curative radiation and concurrent LDC (40 mg/m^2^ weekly) for p16-positive OPSCC from January 2015 to December 2021 were retrospectively analysed (*n* = 103). Primary endpoints included overall survival (OS) and progression-free survival (PFS), with secondary endpoints being loco-regional and distant recurrence rates, and chemotherapy-related toxicity. Results: Overall survival rates were 94%, 89%, and 84% at two, three, and five years, respectively. The progression-free survival rates were 90%, 83%, and 80% over the same periods. The low-risk group showed excellent outcomes with no loco-regional recurrences and low distant relapse rates (6% at 5 years). The intermediate-risk group had higher loco-regional recurrences (10% at 5 years) and a similar distant relapse rate (7% at 5 years). T4 or N3 disease patients had lower survival and higher distant relapse rates but excellent loco-regional control. The median cumulative cisplatin dose was 240 mg/m^2^, with no survival difference between those receiving ≥200 mg/m^2^ versus less. Toxicity led to dose reductions (8%), delays (13%), and early cessations (30%). Conclusions: LDC is effective for p16-positive OPSCC with manageable toxicity, showing outcomes comparable to outcomes previously reported in studies using HDC.

## Simple Summary

P16-positive oropharyngeal squamous cell carcinoma has an increasing incidence with a relatively good response to treatment compared to non-HPV-related cancers. Locally advanced OPSCC is managed with chemoradiation—standard of care being high-dose cisplatin, which is associated with significant toxicity. This study aims to look at the outcomes at a single regional centre using low-dose cisplatin, which has been shown to be of comparable efficacy with better tolerability in the adjuvant setting and emerging evidence in the definitive setting. Our findings show comparable outcomes compared to those reported in the literature using high-dose cisplatin, supporting further study into this as an alternative treatment approach.

## 1. Introduction

The incidence of p16-positive oropharyngeal squamous cell carcinoma (OPSCC) continues to increase with approximately 21,000 new cases in 2020 in the US [1]. In Queensland, Australia, OPSCC is now the most commonly diagnosed mucosal head and neck cancer, with the majority being HPV-associated as indicated by p16 positivity [2]. It is a clinically distinct disease from non-HPV-related head and neck squamous cell carcinoma, and has a better prognosis [3]. As a result of the increasing number of survivors, there has been investigation into de-escalation of therapy including systemic therapy to reduce long-term effects.

Current standard of care for locally advanced OPSCC includes concurrent high-dose cisplatin (HDC; 100 mg/m^2^ given every 3 weeks) with radiation therapy [4,5]. However, there is a significant increase in mucositis, ototoxicity, nephrotoxicity, and haematological toxicity with this approach [6]. Additionally, studies have highlighted that nearly 40% of patients are unable to receive the final dose, with a cumulative dose of 200 mg/m^2^ deemed sufficient for effect [7,8].

To minimise toxicity while preserving efficacy, there is increasing interest in alternative low-dose weekly cisplatin regimens (LDC). Kiyota et al.’s randomized controlled trial showed similar relapse-free and overall survival outcomes when comparing LDC (40 mg/m^2^ every 1 week) versus high-dose cisplatin (HDC; 100 mg/m^2^ every 3 weeks) concurrent with radiation in the adjuvant setting for high-risk resected locally advanced head and neck squamous cell carcinoma (LAHNSCC) [9]. In the definitive setting, emerging results from the ConCERT trial also showed non-inferior outcomes of LDC [10]. Similarly, a meta-analysis of several prospective trials indicated no survival differences between LDC and HDC in the framework of conventional fractionated radiation [11]. However, there remains clinical uncertainty as to whether it should be standard of care in the definitive setting. In low-risk p16-positive OPSCC, excellent progression-free and overall survival outcomes with weekly LDC (40 mg/m^2^ given every week) were demonstrated in the TROG 12.01 trial [12].

The Townsville Cancer Centre (TCC), situated in North Queensland, is a regional cancer care facility that provides oncology services to its local health district and rural satellite centres. TCC provides the only head and neck service for North Queensland, therefore covering a catchment extending from Mackay to the Torres Strait, and west to the Northern Territory border (approximately 700,000 people) [13]. Unique qualities of providing a head and neck service in a regional area include the higher frequency of treatment of patients residing in rural or remote regions (defined as town population of less than 50,000 people as per Modified Monash Model), and Aboriginal and Torres Strait Islander people (indigenous peoples of Australia) [14].

In our institution, we have exclusively used LDC (40 mg/m^2^ every 1 week) for cisplatin-eligible patients undergoing definitive chemo-radiotherapy (CRT) for p16-positive OPSCC. Therefore, this study aims to provide unique insight into real-world data on outcomes with low-dose weekly cisplatin in p16-positive OPSCC, in the absence of selection bias for high-dose cisplatin. Moreover, it aims to highlight outcomes in a regional centre, and Aboriginal and Torres Strait Islander patients.

## 2. Methods

### 2.1. Study Design and Patient Population

This was a retrospective review of a prospective head and neck cancer database and electronic patient records. Patients were selected from the database if they were treated with curative-intent radiotherapy with concurrent LDC (40 mg/m^2^ given every week) for p16-positive oropharyngeal squamous cell carcinoma at Townsville University Hospital from January 2015 to December 2021. P16 status was used as a surrogate for human papillomavirus (HPV) performed via immunohistochemical staining. Curative-intent treatment was defined as an external beam radiotherapy plan with a biological equivalent dose (BED) of at least 66 Gy in 2 Gy fractions to all gross disease delivered via volume-modulated arc technique. 93 (90.3%) patients received 70 Gy in 35 fractions, seven (6.8%) patients received 69.96 Gy in 33 fractions, and three (2.9%) patients stopped treatment early after receiving BED of at least 66 Gy in 2 Gy fractions. Treatment of elective nodal regions was based on international guidelines with regard to the primary site and clinician’s judgement. The prophylactic dose is covered by BED of 50 Gy in 2 Gy fractions [15]. There was no change to radiation protocol over the study period. The intended chemotherapy regimen was weekly cisplatin 40 mg/m^2^ given on Weeks 1, 2, 3, 4, 5, and 6 concurrent with radiation to a planned cumulative dose of 240 mg/m^2^. Cisplatin was given on week 7 if there were dose delays during weeks 1 to 6. For patients who did receive the intended regimen, there was variable use of cisplatin on week 7 based on clinician preference. The cumulative dose of cisplatin administered was recorded (as mg/m^2^). The study received institutional ethics-waiver approval from the Human Research Ethics Committee at Townsville University Hospital, on the grounds of retrospective audit.

### 2.2. Risk Stratification and Patient Follow-Up

Data was collected using electronic medical records. Demographic data recorded included age, gender, Eastern Cooperative Oncology Group performance status (ECOG), smoking history (greater or less than 10 pack-year history), rurality (rural defined as Modified Monash categories 3–7) [16], Aboriginal or Torres Strait Islander (Yes or No), and TNM stage according to the American Joint Committee on Cancer 7th edition [17]. Patients were stratified as low risk as per Ang classification, if they had AJCC 7th edition N0 to 2a disease, or N2b to 3 disease and a smoking history of <10 pack years. Intermediate risk was defined as N2b to 3 disease and a smoking history of ≥10 pack years [18]. There was no high-risk group as p16-negative or unknown patients were excluded. Clinical assessment was routinely performed every 3 months for the first 2 years, every 4 months in years 3 and 4, and every 6 months in year 5. Radiological follow-up was performed at 3 months (FDG PET/CT) post-completion of chemo-radiotherapy, and repeat imaging based on whether there was residual FDG avidity or ongoing structural abnormality of concern on CT.

### 2.3. Study Outcomes

The primary end points were overall survival (OS) and progression-free survival (PFS). OS was measured as the time from starting radiation therapy to death from any cause. PFS was measured as the time from the initiation of radiation therapy to the development of loco-regional recurrence, distant recurrence, or death. Loco-regional recurrence was defined as recurrence at primary site or regional nodal stations, while distant recurrence was defined as metastatic disease outside the prior sites. It should be noted that if patients were lost to follow-up prior to progression, they were censored at the date they were lost to follow-up, even if they later passed away. If their date of death was known, this was used to calculate overall survival. The secondary outcomes were loco-regional recurrence rate, distant recurrence rate, cumulative cisplatin dose, and toxicity that led to a reduction in dose, dose delays, or premature discontinuation of cisplatin. The omission of cisplatin in the seventh week for patients who had already completed six cycles of weekly administration was not categorized as early cessation, given the variability in its usage among clinicians.

### 2.4. Statistical Analysis

Data management was conducted using Microsoft Excel. Baseline characteristics were summarized using descriptive statistics including counts and frequencies for categorical variables and mean, standard deviation (SD), median, and range for continuous variables. Kaplan–Meier survival analysis was used to calculate PFS and OS. The differences between groups were evaluated using the log-rank test. The PFS and OS were also compared between risk groups with a Cox regression model—given the limited number of events (16 deaths and 19 progression events), multivariable analysis was not completed. All statistical analyses were performed in STATA version 18.

## 3. Results

### 3.1. Demographics

**Figure 1:**
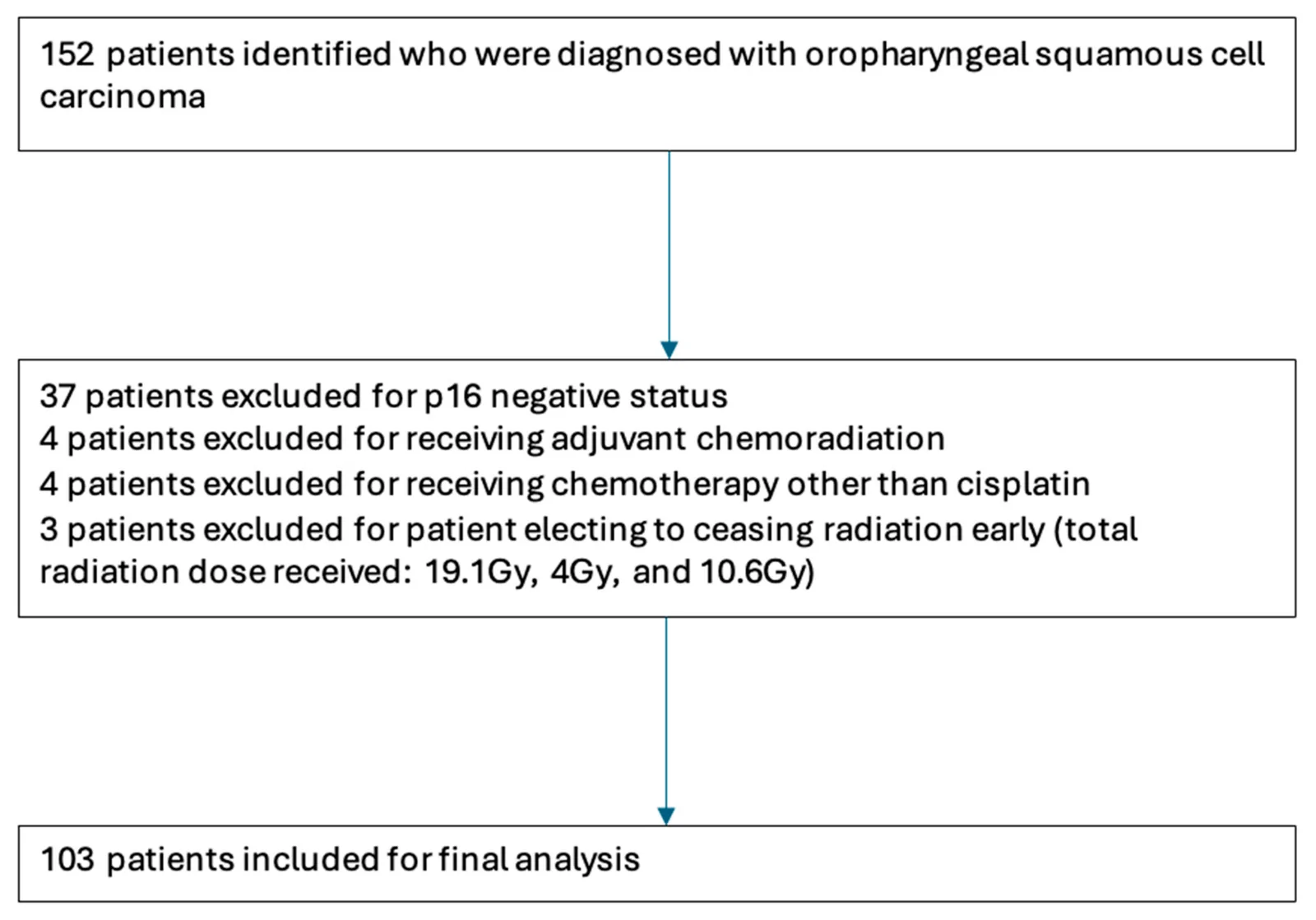
Patient selection diagram.

We identified 152 patients who were diagnosed with oropharyngeal squamous cell carcinoma for the study (Figure 1). A total of 49 patients were excluded; criteria for exclusion included p16-negative or unknown (*n* = 37; 24%), receiving chemo-radiation in the adjuvant setting (*n* = 4; 3%), receiving concurrent chemotherapy other than cisplatin (*n* = 4; 3%), and metastatic disease at diagnosis (*n* = 1; 1%). Three patients (2%) were excluded from analysis for ceasing radiation therapy early due to patient choice (total radiation dose received: 19.1 Gy, 4 Gy, and 10.6 Gy). The remaining 103 patients were included for final analysis.

Demographics are shown in Table 1. The most common primary site of malignancy was tonsil (*n* = 66; 64%) followed by base of tongue (*n* = 31; 30%). 38 patients (37%) had T3 or T4 disease, while 90 patients (87%) had N2 or N3 disease. 48 patients (47%) had a smoking history of less than 10 pack-years. Five patients (5%) were Aboriginal or Torres Strait Islander. Most patients were ECOG 0 to 1 (98%). 75 patients (73%) resided in rural or remote areas at the time of treatment. 57 patients (55%) were defined as having low-risk disease, while 46 patients (45%) had intermediate-risk disease. Three patients (3%) did not complete planned radiation because of toxicity (*n* = 3; range 60–62 Gy). The median dose administered was 70 Gy (Range: 60–70), and median number of fractions was 35 (Range: 30–35).

### 3.2. Overall Efficacy

**Figure 2:**
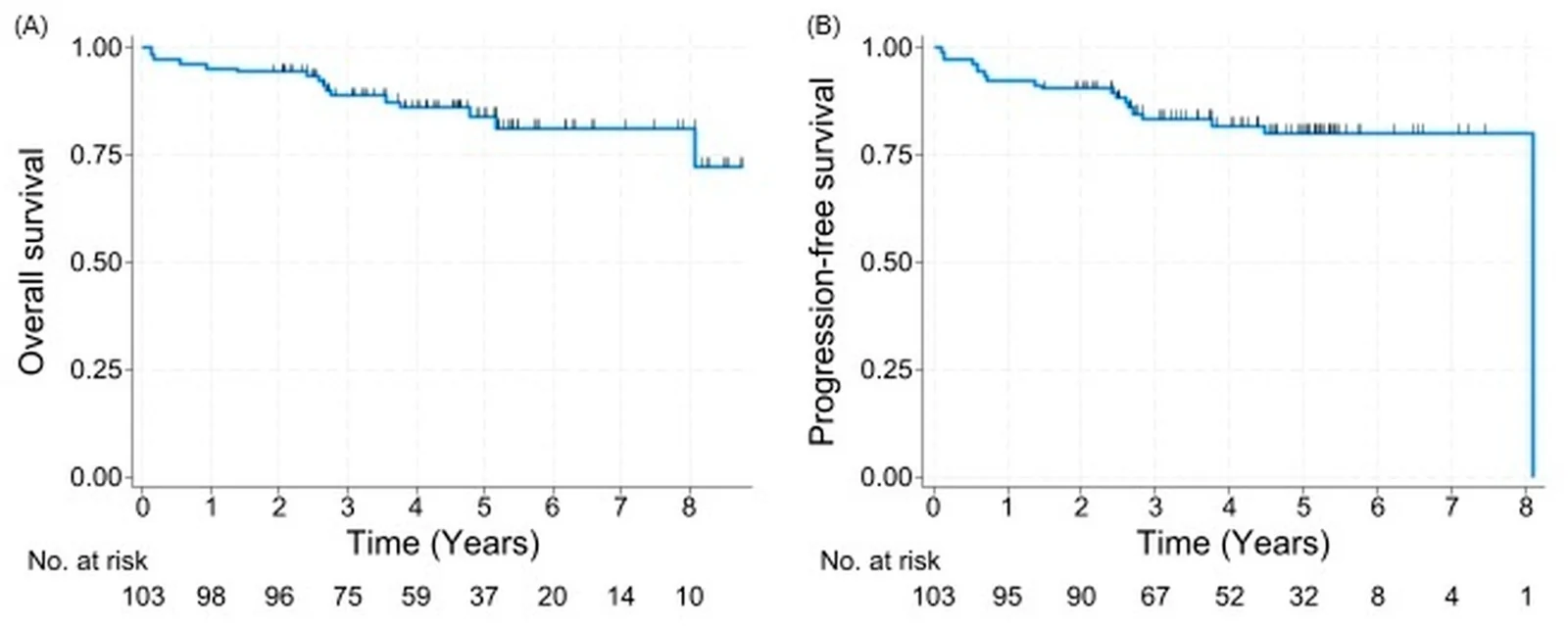
Kaplan–Meier curve illustrating (**A**) overall survival and (**B**) progression-free survival of the entire cohort.

The median follow-up time was 56 months. The overall median survival was not reached (NR). The two-, three-, and five-year overall survival data with 95% CIs were 94% (90%, 99%), 89% (82%, 95%), and 84% (76%, 92%), respectively (Figure 2A). The median progression-free survival was 98.5 months (NA, NA), with two-, three-, and five-year progression-free survival of 90% (85%, 96%), 83% (76%, 91%), and 80% (72%, 89%), respectively (Figure 2B). Given the small number of events, the median PFS was affected by a late event, with 95% CIs not calculable. As such, the other estimates are more indicative of the PFS outcomes. Four patients (4%) had loco-regional recurrence (one patient with local recurrence and three patients with regional nodal recurrence), with two patients subsequently undergoing salvage procedures (one patient undergoing salvage neck dissection for residual regional nodal disease with malignancy identified, and the other salvage neck dissection for recurrent regional nodal disease post initial complete response). Five patients (5%) had distant metastatic recurrence. No patients had first relapse of both loco-regional and distant concurrently.

### 3.3. Low-Risk Subgroup

**Figure 3:**
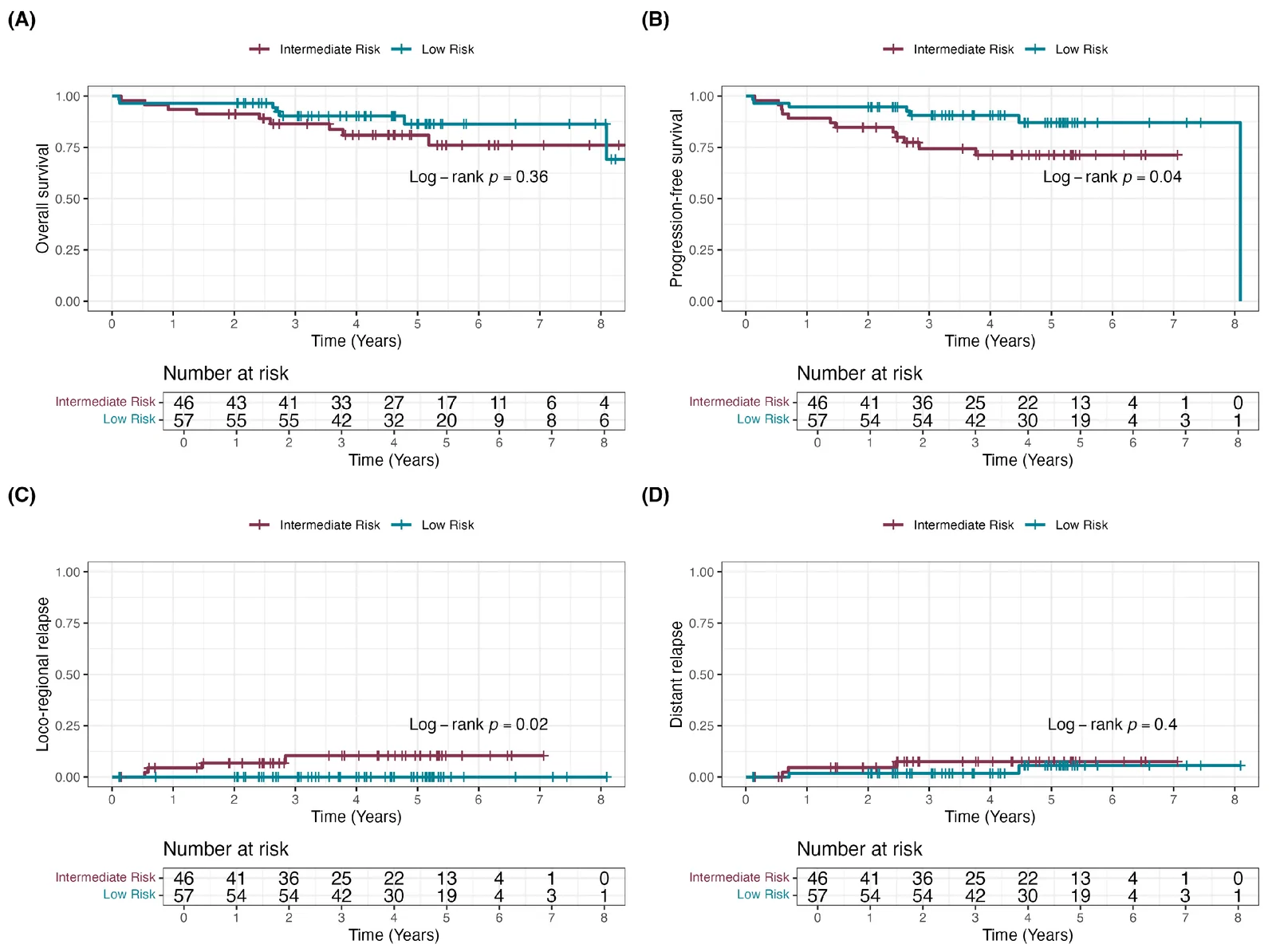
Kaplan–Meier curve illustrating (**A**) overall survival, (**B**) progression-free survival, (**C**) loco-regional relapse, and (**D**) distant relapse of low-risk p16-positive OPSCC versus intermediate-risk p16-positive OPSCC.

57 patients were included in the low-risk group, with nine patients (16%) having a smoking history of more than 10 pack years. The median overall survival was not reached. The two-, three-, and five-year overall survival rates were 97% (92%, 100%), 90% (83%, 99%), and 86% (76%, 98%), respectively (Figure 3A). Similarly, the median progression-free survival was 98.5 months, with corresponding two-, three-, and five-year rates of 95% (89%, 100%), 91% (83%, 99%), and 87% (78%, 98%) (Figure 3B). There were no cases of loco-regional recurrence, while distant relapses occurred in two patients, resulting in two- and five-year distant failure rates of 2% and 6% (Figure 3C,D).

### 3.4. Intermediate-Risk Subgroup

46 patients were included in the intermediate-risk group. The median overall survival and median progression-free survival were not reached. Compared to the low-risk group, there was a worse overall survival, with lower two-, three-, and five-year rates of 91% (84%, 100%), 87% (77%, 97%), and 81% (70%, 94%), respectively (Low- versus intermediate-risk group, log-rank *p* = 0.36; Figure 3A). Comparing the two groups’ overall survival using a Cox regression model found a hazard ratio of 0.63 (95% CIs 0.24–1.71, *p* = 0.36)—indicating the low-risk group had lower mortality but no statistically significant association. Given the small number of total deaths, subgroup analysis is only exploratory. Progression-free survival was also lower in this group, with two-, three-, and five-year rates of 85% (75%, 96%), 74% (62%, 89%), and 71% (59%, 87%), respectively (Low- versus intermediate-risk group, log-rank *p* = 0.04; Figure 3B). Comparing the two groups’ progression-free survival found a hazard ratio of 0.37 (95% CIs 0.14–0.99, *p* = 0.04). This is in accordance with the log-rank test indicating significantly reduced progression in the low-risk group—noting that there was a low number of censoring events (19 total), which results in the wide confidence interval. Loco-regional relapses were more frequent, with four reported cases and higher two-, three-, and five-year loco-regional failure rates of 7%, 10%, and 10% (Low- versus intermediate-risk group, log-rank *p* = 0.02; Figure 3C). Distant relapses occurred in three patients, leading to two-, three-, and five-year distant failure rates of 5%, 7%, and 7% (Low- versus intermediate-risk group, log-rank *p* = 0.4; Figure 3D).

### 3.5. T4 or N3 Oropharynx Subgroup

**Figure 4:**
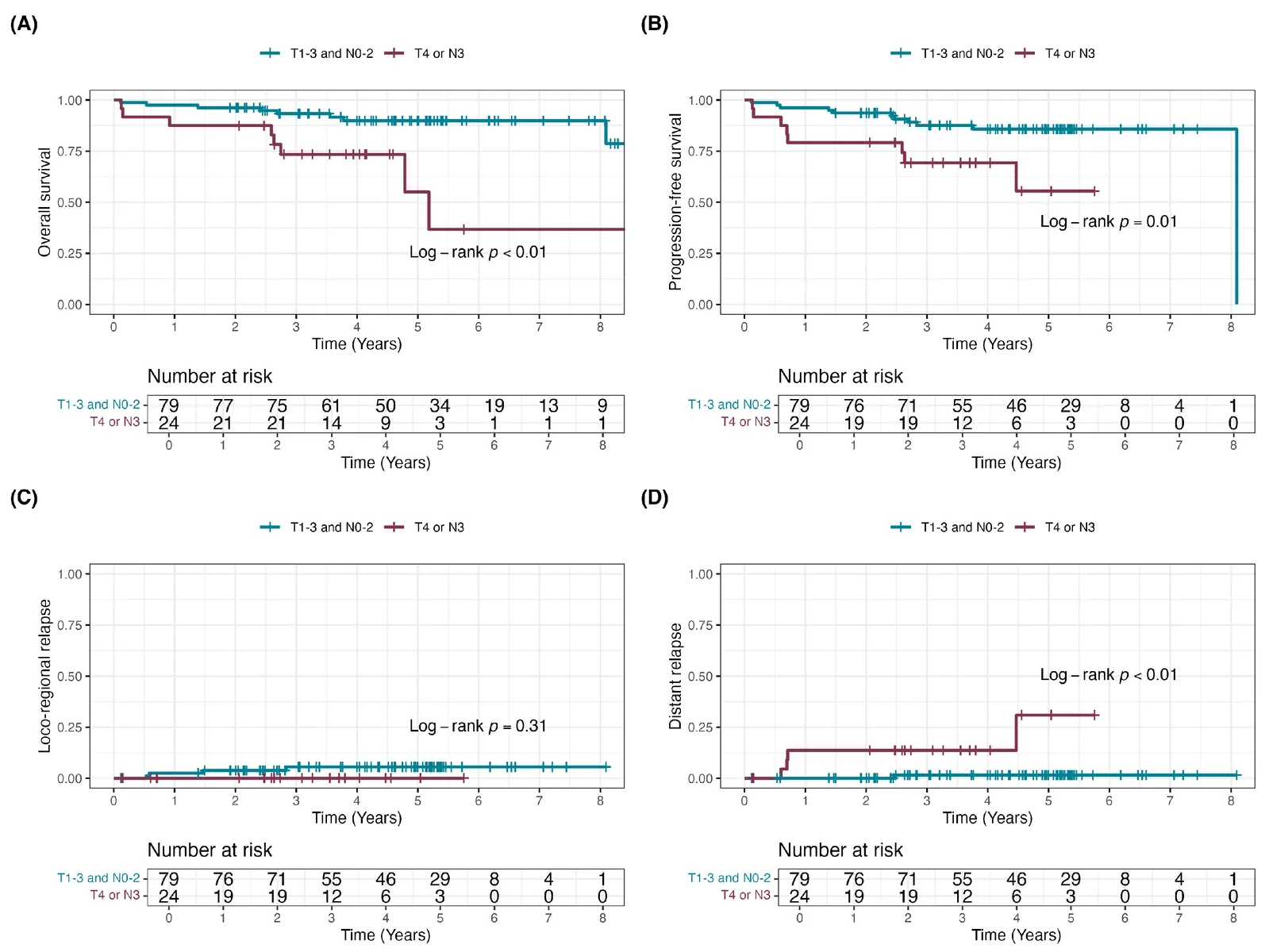
Kaplan–Meier curve illustrating (**A**) overall survival, (**B**) progression-free survival, (**C**) loco-regional relapse, and (**D**) distant relapse of T4 or N3 p16-positive OPSCC versus T1–3 and N0–2 p16-positive OPSCC (AJCC 7).

There were 24 patients (23%) with T4 or N3 disease. 19 patients had T4-only disease, three patients had N3-only disease, and two patients had T4 and N3 disease. 11 patients (46%) had a smoking history of less than 10 pack years and were defined as low risk, while 13 patients (54%) were defined as intermediate risk. Overall survival was significantly lower in comparison to the rest of the cohort (median 63 months v NR; log-rank *p* < 0.01), as was progression-free survival (median NR v NR; log-rank *p* = 0.01). Cox regression modelling found a hazard ratio of 4.36 (95% CIs 1.61, 11.8; *p* = 0.0038) for overall survival and 3.19 (95% CIs 1.25, 8.13; *p* = 0.015) for progression-free survival. Two-, three-, and five-year overall survival rates were 88% (75%, 100%), 73% (57%, 94%), and 55% (30%, 100%), respectively (Figure 4A) and two-, three-, and five-year progression-free survival rates were 79% (64%, 97%), 69% (53%, 91%), and 55% (33%, 93%) (Figure 4B). However, loco-regional disease control was excellent in this group, with no loco-regional relapse (Figure 4C). Distant relapse rate was higher (*n* = 4; 17%) with two-, three-, and five-year failure rates of 14%, 14%, and 31% (log-rank *p* < 0.01; Figure 4D).

### 3.6. Aboriginal or Torres Strait Islander Subgroup

Five Aboriginal or Torres Strait Islander patients were treated in this cohort. This included three patients with T4 or N3 disease. Median overall survival and progression-free survival were not reached. There were no loco-regional or distant relapses noted. Four patients were still alive at the time of analysis, with one patient’s death of unclear cause during treatment.

### 3.7. Cisplatin Dose Correlation

The cumulative median dose of cisplatin administered was 240 mg (IQR: 200–240 mg). 83 patients (81%) received a cumulative cisplatin dose of at least 200 mg, and 65 patients (63%) received at least 240 mg. All Aboriginal or Torres Strait Islander patients achieved a cumulative cisplatin dose of at least 200 mg. Analysis of the entire cohort found no statistically significant association in OS between patients who received 200 mg/m^2^ or more versus those who received less than 200 mg/m^2^ of cisplatin (5-year OS of 86% v 78%, *p* = 0.76) or in PFS (5-year PFS of 86% v 77%, *p* = 0.67) (Table 2). Cox regression modelling comparing ≥200 mg/m^2^ to <200 mg/m^2^ found a hazard ratio of 0.84 (0.27–2.61, *p* = 0.763) for OS and 0.98 (0.32–2.96, *p* = 0.97) for PFS.

### 3.8. Toxicity Related to Chemotherapy

Overall, eight patients (8%) required a dose reduction of cisplatin, 13 patients (13%) required a dose delay, and 31 patients (30%) required early cessation of the chemotherapy regimen. Table 3 highlights the toxicity profile. The most common cause of dose reduction was elevated creatinine (4%). Causes for dose delays included elevated creatinine (3%), mucositis (3%), and non-neutropenic infections (2%). Common reasons for early cessation of chemotherapy were thrombocytopenia (7%), mucositis (7%), and neutropenia (6%). There was one death due to unclear cause during treatment.

## 4. Discussion

This retrospective study assessed clinical outcomes in patients undergoing LDC concurrent with radiation in p16-positive OPSCC. Importantly, our centre exclusively used LDC, based on efficacy noted on prior analysis at the same centre, reducing any selection bias [19]. Overall, it showed similar outcomes to landmark clinical trials which used HDC in this population in a real-world setting [20]. RTOG 1016 was a de-escalation trial to assess the impact of cetuximab versus HDC in p16-positive OPSCC concurrent with RT. 5-year overall survival in the HDC arm was 84.6%, progression-free survival was 78.4%, and loco-regional relapse rate was 9.9% [20]. Despite having a smaller proportion of low-risk patients (55% in our cohort compared to 71% in RTOG 1016), our outcomes appear similar with LDC [20].

In the low-risk subgroup in our study, there was no loco-regional relapse, infrequent distant relapse, and a 5-year overall survival of 86%, supporting positive outcomes with LDC. Combined analysis of the OPSCC low-risk subgroups of RTOG 0129 and RTOG 0522, which both used HDC concurrent with radiation, showed a loco-regional failure rate of 11% and similar overall survival at 5 years [21]. The HDC control arm of the De-ESCALaTE HPV trial also showed similar outcomes to our study with two-year overall survival of 98% and recurrence risk of 6% [22]. LDC was used as the control arm in the randomised TROG 12.01 trial, which recruited low-risk p16-positive OPSCC but excluded T4 and N3 disease, showing three-year overall survival of 98% and three-year failure-free rate of 93% [12]. In our study, this subgroup had no loco-regional relapse, no distant relapses, and an overall survival of 95% at 5 years, supporting the benefit of LDC in this group, similar to outcomes noted in the TROG 12.01 trial.

In the intermediate-risk group, five-year overall survival was 81% in this study, lower than the low-risk group although not statistically significant (*p* = 0.36). There was a higher rate of loco-regional relapse of 10% at 5 years. Combined subgroup analysis of RTOG 0129 and 0522 of the intermediate-risk group showed five-year survival of 66% and loco-regional relapse rate of 15% [21]. Similar loco-regional relapse rate and overall survival to landmark trials were reassuring for similar efficacy to HDC and for ongoing use of LDC in this setting. The highest-risk group was also taken into consideration, defined as T4 or N3 disease. There was no loco-regional relapse in this cohort. Given its small size, definite conclusions cannot be drawn; however, it supports further study into the efficacy of LDC in this cohort. Overall survival outcomes were worse than the T1–3 or N0–2 subgroup, which could be attributed to higher distant relapse risk (31% at 5 years) and death from other causes. It is unclear whether HDC would have reduced distant relapse rates in this setting. Improvement in outcomes with locally advanced head and neck cancer with CRT with HDC over radiation alone was predominantly from improving local and regional control, with no impact on distant recurrence [7,8]. Similarly, in p16-positive OPSCC, RTOG 1016 did not show a reduction in distant recurrence rate with HDC when compared to cetuximab [20]. On the contrary, in low-risk p16-positive OPSCC, both TROG 12.01 and De-ESCALaTE HPV showed reduction in distant recurrence rate with LDC and HDC in comparison to cetuximab, respectively. However, both approaches appeared to reduce distant recurrence to a similar degree [12,22].

Similar efficacy between LDC and HDC in LAHNSCC has been shown in prospective trials, although in the adjuvant radiotherapy setting. Kiyota et al. recruited post-operative high-risk LAHNSCC (predominantly oral cavity [46%] and hypopharynx [34%]), eligible for chemoradiotherapy, and compared HDC (100 mg/m^2^ every 3 weeks) to LDC (40 mg/m^2^ every 1 week) with results showing similar 2-year overall survival and progression-free survival [9]. Similarly, emerging results from the ConCERT trial, which recruited LAHNSCC for definitive chemoradiotherapy again comparing HDC (100 mg/m^2^ every 3 weeks) to LDC (40 mg/m^2^ every 1 week), showed non-inferior outcomes with similar 2-year overall and progression-free survival [10]. The majority of patients had p16-negative oropharyngeal cancer. Noronha et al. randomized locally advanced head and neck SCC to 100 mg/m^2^ of 3-weekly cisplatin or 30 mg/m^2^ of weekly cisplatin with radiation. It primarily recruited oral cavity SCC (87%), and 93% were treated in the adjuvant setting. On the contrary, it showed inferior loco-regional control with weekly cisplatin, although this could be attributed to the lower dose of cisplatin use, suggesting 40 mg/m^2^ is necessary for weekly treatment [23]. A large international randomised controlled trial (NRG-HN009) has recently closed to recruitment to further delineate if LDC is non-inferior to HDC in the definitive setting [24].

Reassuringly, 81% of patients in our study were able to receive a cumulative dose of 200 mg/m^2^ or greater (target dose deemed sufficient for effect), and median cumulative dose was 240 mg. In TROG 12.01, median cumulative cisplatin dose was also 240 mg; however, a higher percentage of patients (91%) achieved a dose of 200 mg/m^2^ or greater, while in the ConCERT trial 81.6% achieved this dose [10,12]. It is reassuring that in a regional centre, cumulative dose of cisplatin using LDC was comparable to trial populations. Cumulative dose of ≥200 mg/m^2^ of cisplatin had no statistically significant difference in outcomes compared to <200 mg/m^2^, although this has been highlighted in p16-positive oropharyngeal primary SCC previously [25]. It should be noted that the small size of the <200 mg/m^2^ cohort may have impacted this lack of statistical significance between outcomes. Dose delays or early cessation were as expected with acute kidney injury, cytopenia, and mucositis.

Historically, head and neck cancer outcomes in regional and rural areas of Australia have been worse compared to metropolitan populations, as demonstrated by previous studies exploring the impact of remoteness on patient outcomes [26]. In contrast, the present study demonstrates that similar outcomes to those reported in landmark trials can be achieved in a regional centre despite the majority of the cohort being from rural and remote areas. Furthermore, disparities in outcomes for First Nations patients have been well documented, with poorer results compared to the broader population [27]. Although a small subgroup, this study suggests that with definitive therapy utilising LDC, outcomes for p16-positive oropharyngeal cancer can be comparable to those observed in the broader population. The proportion of First Nations patients in this cohort was lower than anticipated, which likely reflects the absence of the rising incidence of p16-positive oropharyngeal cancer seen in the general population, a trend not yet observed among First Nations people [28].

Limitations of this study include its retrospective nature and the lack of comparison to a high-dose cisplatin arm. Moreover, cross-trial comparison has inherent limitations in interpretation, given variation in protocols [29]. However, the outcomes identified being similar to landmark trials are reassuring for ongoing use at our centre and bring it into consideration for wider practice. Lower-grade toxicity outcomes can also be under-represented considering the retrospective nature, although significant toxicity that did lead to the modification of the regimen was recorded accurately [30]. Lastly, due to weekly dosing, this approach requires more chemotherapy day-unit service time and resources, which may not be feasible for all centres.

## 5. Conclusions

In conclusion, LDC appears to be efficacious in the management of locally advanced p16-positive OPSCC with manageable toxicity. Moreover, treatment in a regional centre can result in similar outcomes to landmark clinical trials in rural, remote, and Aboriginal and Torres Strait Islander Australians.

## Figures and Tables

**Table 1:** Demographics of patient cohort.

Demographics	*n*	%
Median Age (range)	60 (36–76)	
Sex		
M	97	94.2%
F	6	5.8%
Rural		
Yes	75	72.8%
No	28	27.2%
Indigenous		
Yes	5	4.9%
Pack years		
<10	48	46.6%
≥10	55	53.4%
ECOG		
0	55	53.4%
1	46	44.7%
2	2	1.9%
3	0	0.0%
4	0	0.0%
Primary		
Tonsil	66	64.1%
Base of Tongue	31	30.1%
Other	6	5.8%
Stage		
T0	0	0.0%
T1	16	15.5%
T2	49	47.6%
T3	17	16.5%
T4a	19	18.4%
T4b	2	1.9%
N0	5	4.9%
N1	8	7.8%
N2a	8	7.8%
N2b	58	56.3%
N2c	19	18.4%
N3	5	4.9%
Stage		
I	1	1.0%
II	1	1.0%
III	9	8.7%
IVA	85	82.5%
IVB	7	6.8%

**Table 2:** Overall and progression-free survival based on cumulative cisplatin dose ≥ 200 mg/m^2^ or <200 mg/m^2^; CI: Confidence Intervals.

**Overall Survival**				
**Duration**	**Cumulative Dose of Cisplatin ≥ 200 mg/m^2^ (95% CIs)**	**Number at Risk/Number of Events**	**Cumulative Dose of Cisplatin < 200 mg/m^2^ (95% CIs)**	**Number at Risk/Number of Events**
**2 year**	0.939 (0.889, 0.992)	76/5	0.952 (0.866, 1)	20/1
**3 year**	0.881 (0.811, 0.958)	58/4	0.902 (0.782, 1)	17/1
**5 year**	0.863 (0.787, 0.947)	29/1	0.775 (0.600, 1)	9/2
**Progression-free survival**				
**Duration**	**Cumulative Dose of Cisplatin ≥ 200 mg/m^2^ (95% CIs)**	**Number at Risk/Number of Events**	**Cumulative Dose of Cisplatin <200 mg/m^2^ (95% CIs)**	**Number at risk/Number of Events**
**2 year**	0.926 (0.871, 0.985)	70/9	0.952 (0.866, 1)	20/1
**3 year**	0.879 (0.808, 0.957)	51/4	0.852 (0.711, 1)	16/2
**5 year**	0.858 (0.778, 0.946)	26/1	0.767 (0.583, 1)	9/1

**Table 3:** Chemotherapy-related toxicity leading to cessation, dose delay, or dose reductions (note some patients had multiple toxicities causing cessation/delay/reduction).

	Cessation		Dose Delay		Dose Reduction	
	n	%	n	%	n	%
Mucositis *	7	7%	3	3%	0	0%
**Thrombocytopenia**	7	7%	1	1%	0	0%
**Neutropenia**	6	6%	0	0%	1	1%
**Creatinine**	5	5%	3	3%	4	4%
**Infection**	5	5%	2	2%	0	0%
**Febrile neutropenia**	3	3%	1	1%	0	0%
**Tinnitus**	2	2%	0	0%	1	1%
**Fatigue**	1	1%	1	1%	1	1%
**Diarrhea**	0	0%	1	1%	0	0%
**Hypotension**	0	0%	1	1%	0	0%
**Rash**	0	0%	1	1%	0	0%
**Nausea**	0	0%	0	0%	1	1%
**Not documented/Other**	1	1%	0	0%	0	0%

* Mucositis likely contributed to by concurrent radiation.

## Data Availability

The datasets in this article are not readily available due to privacy restrictions.
